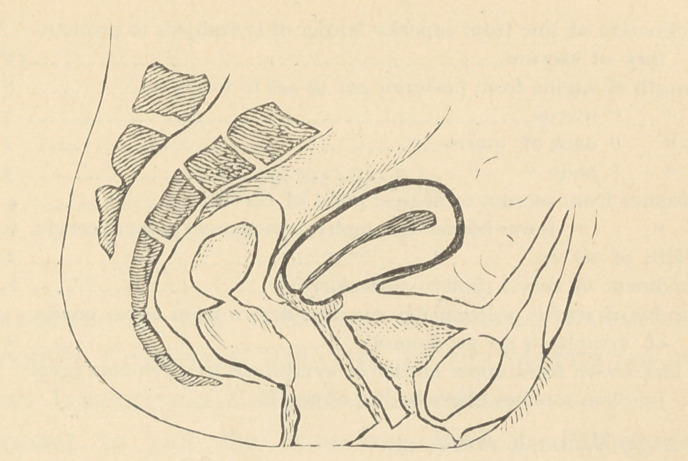# The Normal Position of the Uterus, Etc.

**Published:** 1885-07

**Authors:** Franklin H. Martin

**Affiliations:** 2139 Wabash Avenue; Chicago


THE NORMAL POSITION OF THE UTERUS AND ITS RELATION TO THE OTHER PELVIC ORGANS. *By* FRANKLIN H. MARTIN, M.D., *Chicago.*

*Anatomy.*—I will endeavor in discussing the anatomy of the pelvic organs not to go into unnecessary details, but confine myself to that part of the topography and anatomy of the organs that are subjects of dispute, or, that have been but very recently settled. In considering disputed and unsettled questions, the most that we have a right to do is to take into consideration our own observations, extract from theories, facts, and from the combined evidence impartially draw our conclusions. Among authors of greatest reputation the normal position and condition of the uterus is still a subject of doubt and discussion. To a casual observer it would not seem a difficult matter to settle definitely the true position and relation of the female pelvic organs. During life they admit of the most thorough examination in every way, and after death they are as available for examination apparently as other organs whose topography and anatomy have long been definitely settled.

However, if we take a more critical view of the subject we can discover good reasons for the diversity of opinion that exist. (1) On account of the great mobility of all the parts concerned—the bony pelvis that contains the organs is freely movable upon the long bones of the lower extremities, and the slightest movement here of the pelvis changes the relation of the pelvic organs with the horizontal to just that extent; the natural movements between the pelvis and the spinal column above changes to just that extent the relation of the pelvic organs and the other organs of the trunk. (2) Each organ of the pelvis has functions, the performance of which changes its position and shape. All of these organs are distensible, and as they distend and contract in performing their natural functions, their normal position and relation may constantly vary—if all the pelvic organs are empty and collapsed, they occupy less room, and the vacuum caused by this contraction is filled, if not by the compressible connective tissue surrounding these organs, by the movable organs from above. (3) It is a question whether every respiration of a female does not change the relation of these organs. Every cough or sneeze makes its influence felt upon the relation of these organs to each other and the surrounding parts. (4) A great deal of pains has been taken by anatomists to make frozen sections of the pelvic organs in order to show accurately their relation. Even this has not been a success. It is found that death necessarily changes the relation of these organs—the circulation is stopped, which in life gives the various organs a fullness and erectility; the mucous and serous membranes, the ligaments and connective tissues loose their necessary elacticity; the muscular tissue of the various organs have lost their natural tonicity and are in an unnatural state of relaxation. Therefore, in consequence of these many changes incident to death, a frozen section cannot be entirely free from objections. True, certain devices have been carried out with commendable exactness in detail and earnestness to overcome even some of the objections to frozen specimens, but, as yet, it must be admitted that frozen specimens are not free from objections. (5) At last, if we put aside all other difficulties given above, we find that our methods of examination preclude the possibility of arriving at definite results. A digital examination at once separates the anterior and posterior walls of the vagina, transforms the normally flattened tube into an open cylinder; as we continue our examination, the conjoined manipulation, however carefully done, changes the position or location of the uterus, influences the bladder and compresses the rectum. The most skillful specular examination changes the relation of almost every organ in the pelvis. After considering these few prominent reasons for our ignorance in regard to position of these organs, our uncertainty is not so much a matter of astonishment. There is no doubt, however, but that these difficulties will eventually be overcome by honest perseverance, and a great many of the unsettled questions satisfactorily adjusted, if we carefully consider the accumulated results obtained in the experiments of conscientious observers. At best, in medicine and surgery, it is only by repeatedly verifying clinical results claimed by others, that we are enabled to arrive at definite conclusions. In order to elucidate what seems to us the most rational views on the topography of the pelvic organs, we must first construct a diagram. (See page containing diagram.) The outlines of this diagram should correspond to a lateral view of an anterio-posterior section of the bony frame of the pelvis, and they should be carefully proportioned by a general average of measurements deduced from the tables of measurements of FOSTER and LITZMAN —two authorities who have done conscientious work in unraveling the pelvic problem. Within this outline representing the frame of the pelvis will afterwards be arranged its contents, their position corresponding to the latest views of the best authorities.

*The Pelvic Canals.*—In considering the anatomy of these canals we will take the liberty of quoting AMBROSE L. RANNEY to considerable extent—from his series of excellent articles published in the *American Journal of Obstetrics*, commencing in the March number of 1883.

"The transverse section of the pelvis (first devised by Henle to show the normal outline and relation of the three pelvic canals, viz.: the urethra, vagina and rectum) is now accepted as accurate. It shows that the vagina and rectum are not open tubes, but that they exist when not distended as mere slits in the pelvic section. The long axis of the vaginal slit is transverse, and corresponds, as HART puts it, to the mouth of the woman in its general direction; that of the rectum is directed anterio-posteriorly, thus forming a right angle with the long axis of the vaginal slit; while the urethra appears as a puckered and closed tube." This view, as favored by RANNEY, is not shared by all anatomists and gynecologists, and will be more fully dwelt upon in discussing the rectum.

*The Perineum.*—" This structure seems to be composed of a large excess of elastic tissue intermingled with the muscular fibers of the bulbo-cavernosus, sphincter-ani externus, transversus-perinæi and ischio-coccygeus muscles, and also some fibrous tissue derived from the ischio-perineal ligaments, the deep layer of the superficial perineal fascia, the perineal septum and deep perineal fascia. The result of the fusion of these structures is to produce a body which shall combine a great resistant power with a high degree of elasticity, two elements most essential to this part from its situation and the strain which it is called upon to bear. The perineal body prevents pouching of the anterior wall of the rectum, when the sphincters are contracted. THOMAS attributes to this body the function of a support to both the anterior rectal and the posterior vaginal walls."—(RANNEY.)

*The Vagina.*—" As has been previously stated, this canal appears as a transverse slit in that section of the pelvis devised by HENLE to show the relative position and appearance of the three pelvic canals. In the antero-posterior median section of the pelvis, it appears as a line only and not as an open tube." The vagina does not follow and conform to the concavity of the sacrum as depicted in many of the popular cuts. The coccyx lies above the level of the symphysis pubis when the patient is in the erect position. The vagina extends ordinarily only to about the level of the sacro-coccygeal junction. The vagina does not lie parallel with the sacrum—its line of direction, if extended posteriorly, would cut the sacrum at the third and fourth sacral articulation. It therefore bears no apparent or real relation to the sacrum in regard to position, since it comes in no respect within its limits. Its general course is practically straight; or it may be said to be bent at either extremity, giving it the form of the italic letter *S*. The relation which the direction of the vagina has with the horizontal is yet the subject of considerable dispute among prominent authors. RANNEY is inclined to give it as about 40° with the horizon. HART makes the angle 50°. Scarcely two writers agree upon this point, although the tendency with the later writers is to make it more perpendicular. The anterior and posterior walls of the vagina are normally in opposition.

*The Rectum.*—The rectum is situated between the vagina and the sacrum and coccyx. It does not lie in the median line of the pelvis for its whole extent—being deflected to the right as it descends. It is necessary, therefore, in studying the anatomy and topography of this organ to have this in mind in looking upon an antero-posterior median section of the pelvis. It is necessary in order to appreciate its true relation to view it as depicted in a lateral section. RANNEY says: "Most of the cuts incorporated in the works of the greater anatomists, also in those dealing exclusively with the gynecological department, represent the rectum as piercing the muscular structure of the female pelvic floor obliquely, and reaching to the skin, while the anus is usually depicted as an open tube, affording no apparent obstacle during life to the escape of its contents. It will be perceived, if the observations of others agree with my own, that the thickness of the pelvic floor in this region is about one inch." HART states that the anal canal perforates the muscular structure which forms the pelvic floor in a direction of nearly right angles to the vaginal axis.

The lower part of the normal rectum is usually empty and collapsed. The feces are prevented from occupying this part of the bowel by the semilunar folds of the mucous coat of the rectum called the valves of Houston. These folds are situated three or four inches from the anus and form a constriction at that point. The levator-ani muscle by its anterior fibers, which pass around the outlet of the vagina, form a vaginal sphincter.

*The Uterus.*—We now come to that much talked about organ—the uterus. Many of the best authors writing upon this subject state emphatically that it is impossible to give, with any degree of positiveness, a normal position to the uterus. At the same time these same authors close their chapter invariably with a minute description of the normal position and state of the uterus, and bring argument upon argument to sustain these particular views. A point that is yet a subject of grave discussion is the relation that the uterus sustains to the horizontal, when the woman is in a given position. The older works on anatomy describe and picture the uterus as occupying nearly a perpendicular when the patient is in an upright position—a view which at the present time cannot find recognition for a minute. Following this, and lasting until the very present, comes the other extreme—a position of extreme anteversion and flexion. We have extremes in both directions, and one cannot but think that fashion rules even in the disposition of this important organ To my mind, after weighing the testimony of the best authors on this subject, it is impossible to come to any other conclusion than that we are safer to take neither extreme, but to be satisfied with median ground.

I do not make this statement without being, as I think, prepared to give more convincing reasons for this conclusion than a popular desire to be considered conservative.

SAPPEY maintains that the fundus of the normal uterus lies three-fourths of an inch below the plane of the pelvic brim. RANNEY'S researches convince him that the fundus should be placed slightly above that plane. HART and BARBOUR say that the position of the uterus, with empty bladder and rectum, is such that it lies with its anterior surface touching the posterior aspect of the bladder—no intestines intervening; the cervix uteri looks downwards and backwards, and the uterus is slightly twisted as a whole on its long axis, so that the uterine end of the right Fallopian tube is nearer the spmphysis than that of the left. According to SCHULTZE the uterus is extremely anteverted, the axis of the uterus being nearly horizontal with patient in upright position. FREISCH says the uterus is extremely anteverted, so that the fundus is directed toward the upper margin of the symphysis; the vaginal portion lies about at the point of junction of the sacrum with the coccyx—the entire uterus lying below the pelvic inlet.

After giving thus briefly the views of these authorities it is only doing them justice to mention that they *do* recognize, withal, that the uterus *is* a very *movable organ*, and that it is found normally in many positions. They emphasize that any fixed position would be unphysiological. I will quote CHAMBERLAIN'S language on this, as expressing well the general sentiment in regaad to this point. He says: "The uterus has not *a* normal position, but many normal positions. The norm of the uterus is to be very movable, the most movable of any of the pelvic or abdominal viscera. Its distinctly normal position is that toward which it returns from all its excursions, and in which it rests in the equilibrium of all disturbing forces." The uterus then has not *a* normal position, but it has a point to which there is a tendency for it to return after being influenced in any way from that position. It is very necessary, however, for us as gynecologists, to know approximately where that point is, in order to correct any influence that may be exerted and acting to prevent its returning to that point.

Although we are sometimes inclined to think on account of the deplorable state of health of the great majority of our American women, that nature has not exerted herself as much as she ordinarily does to perfect the organs peculiar to them—that in this particular *at least*, she has laid herself open to criticism—although such *seems* to be the case, our better judgment soon comes in and points out to us the fact, that nature has not been neglectful to woman, but that the difficulty lies in the fact that woman is negligent toward nature. Therefore, in this branch of physiology, as in all others, we will assume that nature endeavors to do her work in the most rational manner.

It is maintained by physiologists of note that the contraction and relaxation incident to the performance of the *normal functions* of the bladder and rectum influence invariably the position of the uterus. Although the uterus is a movable organ and easily influenced in its position, we cannot bring ourselves to think that it is *so created*, this all important *organ*, simply for the accommodation of the rectum and the bladder in their *normal* actions, but, to guard it against external violence and abnormal conditions of the surrounding organs. We *are* obliged to admit *this hypothesis*, however, if we accept the extreme anteversion theory of SCHULTZE, because he represents the anterior surface of the uterus as resting upon the collapsed bladder, and with the body of the uterus in this position the cervix must obviously occupy a position of extreme flexion, or encroach upon the rectal territory.

I believe if the anatomy and physiology of the rectum and bladder are closely studied in reference to their relation to the uterus, that it will be found in their normal workings they do not disturb this organ in the least. We should remember that the uterus at the cervix is supported by the recto-uterine ligaments, or folds of peritoneum which attach it to the sacrum, and prevent an excessive downward or forward displacement of this part of the uterus; opposite this support, and acting in exactly the opposite direction, is the anterior wall of the vagina and the fold of peritoneum (verico-uterine ligaments) which prevent an *excessive upward* and *backward* movement; while the broad ligaments spreading out from either side prevent excessive movements of the uterus laterally, or undue downward displacements The uterus, too, is thoroughly surrounded by loose connective tissue, which assists in preserving its steadiness, and protects it from external influences. This connective tissue supports the uterus especially laterally, filling the spaces between the folds of the broad ligaments, passing down to the floor of the pelvis it forms a lateral supporting cushion to the vagina, which, in turn, by the assistance of the perineum, steadies and supports the uterus from below. We find then, that the uterus is very thoroughly supported without the assistance of the bladder and rectum. In looking closely at the conformation of the rectum and bladder we can readily see, too, how they can contract and relax in performing their normal functions without necessarily encroaching upon the uterus. Whether normally distended or empty they occupy about the same space, as far as influencing the uterus is concerned. The bladder collapses in a triangular shape with the long diameter in the antero-posterior direction; the rectum collapses with the long diameter in the anterior-posterior direction—each occupying at least as much space of the anteriorposterior diameter of the pelvis when collapsed as when normally distended—and, it would require *inordinate abnormal* distention to make them occupy more of this space.

When we recognize so many wise provisions of nature in studying what is positively known in regard to the pelvis, we think that we are justified in assuming that nature has managed the doubtful points in the same wise manner. If such be the case, with our present knowledge of dynamics, we cannot refrain from pointing to the apparent incongruities in such cuts as FRITSCH'S, where extreme anteversion of the uterus is depicted, or on the other hand where the opposite version is pictured. To my mind unless one or the other of these alleged correct positions has actually and conclusively been proved to normally exist, by means of superior methods of examination, there is no rational excuse for the promulgation of such theories.

It would be expected that nature would place the uterus in a position in which it would best be enabled to sustain the weight of the super-incumbent organs and in which the jar and sudden shocks of the body could best be resisted with the woman in the ordinary upright position. But surely, such is not the case if we accept the above extreme views.

The intra-abdominal wave of pressure caused by respiration, coughing or sneezing—impulses given by sudden falls or jars, or even in ordinary locomotion, as it follows the vertical axis of the trunk, strikes first the anterior abdominal wall at a point between the umbilicus and the symphysis pubis, and is immediately deflected in a direction downward and backward into the cavity of the true pelvis. Here we come upon our first grave objection to an extreme anteversion or the opposite extreme—the perpendicular. In extreme anteversion the wave impulse would strike the posterior broad surface of the body of the uterus, and drive it down upon the bladder and anterior wall of the vagina, while in the other position, the perpendicular, the anterior broad surface of the body would receive the impulse to an equal disadvantage, displacing the uterus backward and driving the cervix downward, while if the uterus occupied the position between these two extremes, the narrow crest of the fundus would receive the impulse in the line of the axis of the uterus and the force would become equally distributed through all of its supports. Here, too, the organ would not so directly receive the whole impulse, as it would be equally dispersed upon its sides, the posterior ligaments and anterior supports, and its lateral attachments would receive to equal extent their portion of the impulse.

*Second* :—The manner in which the bladder collapses, to my mind, precludes the possibility, or at least probability, of the uterus occupying normally the position of extreme anteversion. The bladder when collapsed, or when empty is a triangular shaped body—not flat like a plate. The base corresponds to its peritoneal surface, the apex corresponds to the urethra. The posterior or inferior surface corresponds to the anterior wall of the vaginia to which it is intimately attached ; the anterior wall corresponds to the symphysis to which it is loosely attached. It is readily seen, then, that the bladder distends only in the direction of the peritoneum or its one free surface. According to the extreme anteversion theorists the free surface of the bladder and the uterus are in apposition. If such be the case the uterus changes its position constantly as the bladder normally relaxes or contracts—this seems to me very improbable. I believe that this space is usually filled with the light coils of the small intestines.

*Third* :—The broad ligaments receive their external attachments at a point about equidistant from the center of the sacrum posteriorly, and the pubic junction anteriorly, in such a way as to divide the plane of brim of the true pelvis into about equal halves. If the body of the uterus occupies a position in the center of the pelvis on a direct line with the ordinary attachments of these ligaments, which it is at least rational to believe is the case, it occupies a position between the perpendicular of SAVAGE and the extreme anteversion of Fritsch.

*Fourth:*—With extreme anteversion, the cervix, with the fundus occupying a position behind the symphysis, would necessarily have to occupy a position far back in the pelvis, within three-fourths of an inch of the sacrum—with a normal normal conformation of parts this is impossible, without interfering with the rectum.

*Fifth:*—If we take the measurements of FOSTER and LITZMAN into consideration we can at once demonstrate the impracticability of the position given by Savage—the perpendicular. The cervix occupies a position normally at a distance of one and one-half inches from the sacrum, the rectum intervening. It is impossible for the uterus to assume anything like a perpendicular, with the cervix in this position, on account of the anterior curve of the sacrum above, which immediately necessitates an anterior version from the perpendicular of at least fifteen degrees.

I am justified, therefore, I think, in rejecting both the extreme anteversion theory and the perpendicular theory of the position of the uterus, and in doing so I present a schematic drawing that will be found to possess fewer theoretical objections.

Measurements used in constructing the above drawing were taken from Foster and Litzman. They are as follows:

| | |
|---|---|
| Promontory of sacrum to upper border of symphysis | 11.7 c. m. |
| " " " " nearest point " " | 11.0 c. m. |
| " " " " middle of third sacral vertebra | 6.9 c. m. |
| " " " " sacro-coccygeal joint | 10.8 |
| Sacro-coccygeal joint to tip of coccyx | 3.8 |
| Promontory of sacrum to tip of coccyx | 11.6 |
| Highest to lowest point of symphysis | 3.8 |
| Upper border of symphysis to upper border of third sacral vertebra, | 13.5 |
| Lower border of symphysis to sacro-coccygeal joint | 12.5 |
| Lower border of symphysis to tip of coccyx | 9.2 |
| Tip of coccyx to horizontal line touching lower border of symphysis | 2.0 |
| Horizontal distance of angle of sacrum from line falling vertically from promontory | 7.5 |
| Horizontal distance of sacro-coccygeal joint from line falling vertically from promontory | 7.3 |
| Horizontal of tip of coccyx from line falling vertically from promontory | 4.5 |

Additional figures showing the relation of the pelvic organs and their proportions as depicted in above drawing:

| | |
|---|---|
| Inclination of axis of cervix uteri with horizontal | 35° |
| " " " " body of uterus with horizontal | 25 to 30° |
| Vaginal inclination with horizontal | 55° |
| Inclination of line from inferior border of symphysis to tip of coccyx | 12° |

| | | |
|---|---|---|
| Inclination of line from superior border of symphysis to promontory of sacrum | 55° | |
| Length of vagina from posterior *cul de sac* to forchette | 8 | c. m. |
| " " uterus | 7 | c. m. |
| " " neck of uterus | 2 | c. m. |
| " " body " " | 5 | c. m. |
| Distance from sacrum to nearest point of cervix | 4.5 | c. m. |
| " " lower border of symphysis to nearest point of cervix, | 6.3 | c. m. |
| Width of cervix | 2.6 | c. m. |
| Thickness of cervix (antero-posteriorly) | 2.4 | c. m. |
| Fundus of uterus projects above a line drawn from upper border of symphysis to promontory | 5 | c. m. |

A line drawn from upper border of symphysis to sacro-coccygeal junction touches lower border of cervix.

2139 Wabash Avenue.

## Figures and Tables

**Figure f1:**